# Adapting an effective lifestyle intervention towards individuals with low socioeconomic status of different ethnic origins: the design of the MetSLIM study

**DOI:** 10.1186/s12889-015-1343-z

**Published:** 2015-02-12

**Authors:** Dorit Teuscher, Andrea J Bukman, Agnes Meershoek, Reint Jan Renes, Edith JM Feskens, Marleen A van Baak

**Affiliations:** Department of Human Biology, Maastricht University Medical Centre+, NUTRIM School for Nutrition, Toxicology and Metabolism, P.O Box 616, 6200 MD Maastricht, The Netherlands; Division of Human Nutrition, Wageningen University, P.O Box 8129, 6700 EV Wageningen, The Netherlands; Department of Health, Ethics and Society, Maastricht University Medical Centre+, CAPHRI, P.O Box 616, 6200 MD Maastricht, The Netherlands; Division of Strategic Communication, Wageningen University, P.O Box 8130, 6700 EW Wageningen, The Netherlands

**Keywords:** Study protocol, Lifestyle intervention, Health promotion, Socioeconomic status, Ethnic groups

## Abstract

**Background:**

People with low socioeconomic status (SES) and some ethnic minorities are often underrepresented in lifestyle programmes. Therefore, a lifestyle programme was developed especially targeting these groups. Developing this lifestyle programme and designing an intervention study to test the effectiveness of this programme was an informative process in which several obstacles were encountered and choices had to be made. Study protocols, however, rarely describe these obstacles encountered in the protocol design process, and it is not always clear why researchers made certain choices. Therefore, the aim of this article is to describe both the final MetSLIM study protocol and the considerations and choices made in designing this study protocol.

**Methods/Design:**

The developed MetSLIM study has a quasi-experimental design, targeting 30- to 70-year-old adults with an elevated waist circumference, living in deprived neighbourhoods, of Dutch, Turkish or Moroccan descent. The intervention group participates in a 12-month lifestyle programme consisting of individual dietary advice, four group sessions and weekly sports lessons. The control group receives written information about a healthy lifestyle and one group session provided by a dietician. The study contains an elaborate effect, process and economic evaluation. Outcome measures are, among other things, change in waist circumference and the other components of the metabolic syndrome.

**Discussion:**

Matching the preferences of the target group, such as their preferred setting, has implications for the entire study protocol. The process evaluation of the MetSLIM study will provide insight into the consequences of the choices made in the MetSLIM study protocol in terms of reach, acceptability and delivery of the programme, and the effect and economic evaluation will provide insight into the (cost)effectiveness of the lifestyle programme in order to reduce waist circumference among individuals with low SES of different ethnic origins.

**Trial registration:**

Netherlands Trial Register NTR3721 (since November 27, 2012).

## Background

Lifestyle intervention studies have shown that the development of cardiometabolic diseases such as type 2 diabetes mellitus (T2DM) can be prevented or postponed by the combination of a healthy diet and increased physical activity [1,2]. Furthermore, it has been established that certain lifestyle interventions can be cost-effective ways to decrease the burden of cardiometabolic diseases [3]. However, people with low socioeconomic status (SES) and some ethnic minorities are often underrepresented in lifestyle interventions [4-6]. This low level of participation is alarming, since the prevalence of cardiometabolic diseases is especially high in these groups [7-9].

In order to decrease the burden of lifestyle-related morbidity, effective lifestyle interventions are needed for low SES individuals and ethnic minorities. Therefore, a research project was started with the aim of developing a lifestyle intervention study especially targeted at these groups. The project focuses on the adaptation of a lifestyle intervention study named SLIM (Study of Lifestyle intervention and Impaired glucose tolerance Maastricht). The SLIM study was a randomized controlled trial, designed to study in a university setting the effectiveness of a diet and physical activity intervention on glucose tolerance in persons with impaired glucose tolerance [10]. The intervention programme consisted of one hour of individual dietary advice from a dietician every three months; annually, a 90-minute group session from a dietician; and free supervised aerobic and resistance training at the university fitness centre. The SLIM study showed that participants with impaired glucose tolerance improved their glucose tolerance and decreased their diabetes risk by participating in this diet and physical activity intervention [11,12]. Although the effects of the SLIM lifestyle programme were promising, and the programme seemed cost-effective [13], participants with lower SES dropped out earlier than participants with higher SES [12]. Furthermore, as in other studies in the Netherlands, participants had to be fairly fluent in spoken Dutch to be able to participate in the study because all the lifestyle intervention activities and measurements were provided in Dutch only, making it difficult for some ethnic minorities to participate.

The adaptation of the SLIM study protocol into the new study protocol, called MetSLIM, was an informative process in which several choices were made. Study protocols rarely describe obstacles encountered in the protocol design process, and it is not always clear why researchers make certain choices. Transparency, by sharing considerations and choices made, can, however, help other researchers to design their study protocol. Therefore, the aim of this article is to describe the design of the MetSLIM study and the considerations and choices made in adapting the SLIM study protocol to the needs of individuals with low SES of different ethnic origins.

## Methods/Design

The protocol design process was supported by gathering information on the current health status and preferences of the target group; consulting health professionals, researchers and a communications expert; and assessing possibilities in the local community setting. The elements of the original SLIM study protocol and the considerations about maintaining or changing those elements for the MetSLIM study protocol are described in more detail in Tables 1 and 2.

**Table 1:** **From SLIM to MetSLIM: considerations and choices regarding the inclusion and exclusion criteria**

**Criteria**	**Original study protocol: SLIM** [10]	**Considerations**	**Adapted study protocol: MetSLIM**
Inclusion criteria	Mean 2-h blood glucose ≥ 7.8 and ≤12.5 mmol/l	Primary outcome measure of MetSLIM will be waist circumference (see considerations Table 2). There is an on-going discussion about the use of different waist cut-off points for different ethnicities. In order to take height differences between ethnic groups into account, waist-to-height-ratio (WHtR) was chosen as selection criterion [14]. In order to observe a change in waist circumference, only persons with an elevated WHtR will be included [15].	WHtR ≥ 0.5
	Mean fasting blood glucose ≤ 7.8 mmol/l		
	Caucasian	The adapted intervention is aimed at individuals of Dutch, Moroccan or Turkish ethnic origin. Moroccans and Turks are the two largest non-Western ethnic minority groups in the Netherlands [16].	Persons of Dutch, Moroccan or Turkish descent
	Age 40–70 years	In the Netherlands, life expectancy without chronic diseases is 8.9 years lower in women and 10.9 years lower in men among the least educated group compared to the most educated group [17]. Also, the onset of T2DM among the Moroccan and Turkish population living in the Netherlands occurs at an earlier age compared with Dutch people [9]. Apparently, prevention of chronic diseases should start at an earlier age among our target group.	Age 30–70 years
	*No additional inclusion criteria*	As the adapted intervention study is aimed at low SES individuals, the researchers had to investigate where and how they would ideally reach this target group. Commonly used indicators for SES are income, education level and occupation. However, these are individual level indicators and this could create recruitment difficulties. It might be uncommon and illogical for the target group to be selected for lifestyle programmes on the basis of their individual education level, income or occupation. Furthermore, it is more practical to recruit in specific areas and use the postal code as indicator for SES [18]. A disadvantage of using the postal code is that more highly educated persons will also be able to participate in the intervention study. However, the advantage of recruiting in neighbourhoods is that participants will live in the same neighbourhood. This offers us the opportunity to provide all activities close to the participants’ home and to focus on group cohesion and social support in the community.	Living in deprived neighbourhood
Exclusion criteria	Known diabetes mellitus	See consideration WHtR as inclusion criterion.	WHtR ≤ 0.5
	Mean 2-h blood glucose > 12.5 mmol/l		
	Mean fasting blood glucose > 7.8 mmol/l		
	Any chronic disease that makes 5-year survival improbable or that interferes with glucose tolerance, or that makes participation in a lifestyle intervention impossible	Participants must be able to follow a lifestyle programme for one year.	Any mental or physical disability that makes participation in a lifestyle intervention impossible
	Medication know to interfere with glucose tolerance	Since the adapted lifestyle programme aims to decrease waist circumference and improve other factors of metabolic syndrome, persons taking medication for CVD and/or T2DM will be excluded.	Medication for hypertension, hypercholesterolemia, cardiovascular diseases, diabetes mellitus or/and renal failure
	Participants in a regular vigorous exercise programme	In order to measure the effectiveness of the lifestyle programme, participants should not already be participating in other lifestyle programmes.	Participation in another regular vigorous exercise and/or diet programme targeting weight loss
	*No additional exclusion criteria*	Since the minimum age of participants was decreased to women’s fertile years, pregnant or lactating were added as exclusion criteria, as these have an influence on the main outcomes of the MetSLIM study.	Pregnant or lactating

**Table 2:** **From SLIM to MetSLIM: an overview of considerations and choices in the protocol design process**

**Protocol elements**	**Original study protocol: SLIM** [10,12,19]	**Considerations**	**Adapted study protocol: MetSLIM**
Objective	To study whether a diet/physical activity intervention programme can improve glucose tolerance in subjects at high risk of developing type 2 diabetes mellitus.	Because of the overlapping risk factors, the initial idea of the MetSLIM study was to focus on persons with metabolic syndrome (MetS), which is associated with an increased risk both of T2DM and of CVD [20]. However, screening for MetS might impose too high a burden on low SES individuals to participate in the study because of:	To evaluate the effectiveness of an adapted version of the SLIM lifestyle programme to reduce elevated waist circumference and improve other components of the metabolic syndrome in individuals with low socioeconomic status of different ethnic origins.
		- unfamiliarity with MetS	
		- time-consuming screening necessary before potential participants know whether they can actually participate (waiting for laboratory results).	
	*Primary outcome*:	Waist circumference was considered because it is:	*Primary outcome*:
	Change in glucose tolerance (2-h plasma glucose)	- visible for potential participants and therefore easy to communicate	Change in waist circumference
		- one of the components of the metabolic syndrome and a risk factor for cardiometabolic diseases [21].	
Study design	*Setting*:	Distance can be a barrier to participation; target group prefers nearby location, possibly a familiar place. The two universities involved in this study are not located in deprived neighbourhoods. Besides, the number of ethnic minorities living in the cities where the two universities are located is relatively small.	*Setting*:
	At the university		In the community
	*Design*:	RCT design does not seem appropriate because:	*Design*:
	Randomized controlled trial (RCT)	- target group is probably unfamiliar with randomization, which could easily provoke dissatisfaction if participants were randomly allocated to intervention and control group within one community	Quasi-experimental study
		- participants are allowed to bring a friend or family member to different intervention activities (for social support), which could result in spill-over.	
	*Duration*:	The duration of MetSLIM should be shorter given the limited time and budget.	*Duration*:
	4.1 year (range 3–6years)		12 months
Study population	*Inclusion/exclusion criteria:*	See Table 1	*Inclusion/exclusion criteria:*
	See Table 1		See Table 1
	*Recruitment strategies*:	Recruitment strategies should be adapted to needs of target group, taking into account that:	*Recruitment strategies*:
	- Potentially eligible persons from a large existing cohort monitoring health and disease in the general population were approached to participate	- GP is indicated as trustworthy and valued person for the target group [22,23]	- Invitation letter from own GP
		- a personal approach seems to be appreciated	- Personal approach in community centres
	- Through advertisements in the local newspaper	- letterbox drops do not seem to work for this group [24].	
Intervention group	*Nutrition advice*:	Target group preferred group delivery of nutrition advice [25]; therefore group meetings should be added. The topics of the extra group meetings should be related to identified barriers, like financial costs and social occasions [25,26].	*Nutrition advice*:
	- One group meeting a year		- Four group meetings a year, of which one is an introduction/kick-off meeting
	- Four 1-hour sessions of individual advice in one year	The spreading of the four hours of individual advice should be flexible. The involved professionals indicated that they preferred to vary the number and length of consultations to the individual needs of the client. This is in accordance with daily practice.	- Four hours of nutrition advice spread over the year, with regard to the needs of the individual
	*Physical activity lessons*:	Target group indicated that they preferred to be physically active with persons of the same gender [25,27], age and physical condition [25]. Target group indicated that creating a supportive environment can encourage lifestyle change [26].	*Physical activity lessons:*
	- Once or twice a week		- Once or twice a week
	- Provided at the gym on the grounds of the university		- Provided in the community
	- In special SLIM groups		- In special MetSLIM groups
			- Men and women separately
			- Possibility to bring friend or family member
	No participation fee	Some local health professionals preferred a participation fee for participating in the lifestyle programme. Their experience was that persons get used to getting everything for free and will switch to other free programmes once a programme is not free anymore. This could be a problem for the maintenance of programmes. At the same time, the target group indicated that financial cost can be a barrier to a healthy lifestyle, and researchers were concerned about not recruiting enough participants.	No participation fee
Control group	*Activities control programme*:	Because of possible low literacy level of the target group, an information meeting instead of only written materials should be considered.	*Activities control programme*:
	- No additional appointments are scheduled, apart from the annual visits for follow-up measurements		- One group meeting with a dietician about nutrition
	- Participants received oral and written information about the beneficial effects of a healthy diet, weight loss and increased physical activity at the appointment for baseline measurements		- Participants will receive oral and written information about the beneficial effects of a healthy diet, weight loss and increased physical activity (where possible, in their mother tongue)
Measurements	*Physical measurements:*	The measurements were reconsidered taking into account:	*Physical measurements:*
	- Anthropometric measurements	- practical feasibility of doing the measurements at different locations, in the community	- Anthropometric measurements
	- Blood sampling	- possibility of relocating measurements equipment	- Blood and urine sampling
	- Blood pressure	- participants’ unfamiliarity with different measurements.	- Blood pressure
	- Oral Glucose Tolerance Test (OGGT)		
	- 12-lead resting ECG		
	- Incremental exhaustive exercise test on an electronically braked bicycle ergometer		
	*Physical activity:*	Difficulties were expected with filling in diaries because of illiteracy. Additional information should be gathered about determinants of behaviour.	*Physical activity:*
	- SQUASH		- SQUASH
	- 3-day PA record		- Accelerometers
			- Questionnaire on determinants of physical activity
	*Dietary habits:*	Difficulties were expected with filling in diaries because of illiteracy. Additional information should be gathered about determinants of behaviour.	*Dietary habits:*
	- FFQ		- Ethnicity-matched FFQ
	- 3-day food record		- Questionnaire on determinants of healthy diet
	*Quality of life:*	The SF-36 is considered as acceptable to measure quality of life among these populations [28,29].	*Quality of life:*
	SF-36 questionnaire		SF-36 questionnaire
	*Economic evaluation:*	The economic evaluation of a lifestyle programme is important in the context of possible future implementation of the programme. Because it is not known who might be willing to pay for the programme, it is important to consider the costs and effects from different perspectives.	*Economic evaluation:*
	Cost-effectiveness analysis was conducted from a healthcare perspective only [13].		Cost-effectiveness analysis and cost-utility analysis will be done from a societal perspective and a healthcare perspective.
	*Process evaluation:*	Adherence to the nutrition and exercise part of to the lifestyle programme was reported in SLIM [30]. An elaborate process evaluation was lacking however. MetSLIM should include an elaborate process evaluation.	*Process evaluation:*
	Limited data available		Elaborate process evaluation by means of:
			- Researchers’ logbooks
			- Registration forms including an attendance list
			- Non-response survey
			- Drop-out questionnaire
			- Participants’ questionnaire
*Additional considerations*	*Involved staff*:	Staffing should be matched with either ethnicity or gender of the participants, depending on the availability of staff and the needs of participants:	*Involved staff*:
	- Dutch researcher	- fluency of participants’ Dutch language might be low	- Dutch researcher(s)
	- Dutch dietician	- dietician should be able to tailor dietary advice to individuals’ (possibly traditional) eating habits and should be familiar with traditions bound to Islam	- Ethnicity-matched research assistants
	- Sports instructor not gender matched	- gender-matched sports instructors are preferred by some Turkish and Moroccan females.	
			- Ethnicity-matched dieticians
			- Gender-matched sports instructors
	*Language* of information material and questionnaires:	The information materials and questionnaires should be translated because of possible problems with fluency in the Dutch language.	Participants can opt for information in one or more of the following *languages*:
	Dutch		- Dutch
			- Standard Arabic
			- Turkish
	*Receiving results of measurements:*	Participation in health checks seemed to be popular among the target group according to various health professionals; receiving results could help to motivate control group participants to participate in the study’s baseline and final measurements. Apart from the motivational aspect, it is common in healthcare practice that patients are informed about the results of regular blood tests.	*Receiving results of measurements:*
	Yes		Yes

### Objective

The objective of the MetSLIM study is to evaluate the (cost-)effectiveness of the adapted lifestyle programme to reduce elevated waist circumference and improve other components of the metabolic syndrome (MetS) in individuals with low socioeconomic status of Dutch, Turkish and Moroccan origin. It was decided to focus on these groups as they are three of the largest ethnic groups in the Netherlands [16]. Change in waist circumference was chosen as the primary outcome for the study because waist circumference is one of the components of the metabolic syndrome, a risk factor for cardiometabolic diseases [21] and easy to communicate to participants. Secondary outcomes are changes in the other components of MetS (i.e. triglycerides, HDL cholesterol, blood pressure and fasting glucose).

### Study design

The MetSLIM study is a quasi-experimental 12-month intervention study. The intervention study is designed for execution in a community setting. In order to prevent spill-over and to have the opportunity to recruit enough participants, the study will be executed in two cities. Two Dutch cities with sufficient potential to recruit the target population have been identified on the basis of the location of the two involved universities, the presence of low SES neighbourhoods and the number of Turkish and Moroccan citizens [31]. Intervention group participants will be recruited in different neighbourhoods than control group participants. Participants will be measured at baseline and after 12 month. Turkish and Moroccan research assistants will assist in recruitment and data collection for the intervention study. The MetSLIM study is registered in the Dutch Trial Register (NTR3721) since November 27, 2012. The Medical Ethical Committee of Wageningen University approved the study protocol. All participants will give their written informed consent before participating in the study.

### Sample size calculation

The sample size calculation was estimated based on the change in waist circumference as an outcome of SLIM after one year (mean difference between intervention and control group 2.1 cm) [11], and the expectation that we would be able to reach 50% of this effect in a real-life setting among this group. Taking into account a relatively high drop-out rate of 25% compared to the 10% in SLIM [11], we estimated that 252 participants (126 per group) would be required to show this effect with an alpha < 0.05 and 1 – beta > 0.8. The aim is to include equal numbers of male and female participants, equally distributed over the three ethnicities.

### Study population

#### Inclusion and exclusion criteria

Applicants are eligible to participate if they fulfil the following criteria (see Table 1): (1) waist-to-height ratio (WHtR) > 0.5; (2) aged between 30 and 70 years; (3) no medication for high cholesterol, cardiovascular diseases (CVD), T2DM or renal failure; (4) living in a deprived neighbourhood; (5) Dutch, Turkish or Moroccan ethnic origin. Following the definitions of Statistics Netherlands, persons with both parents born in the Netherlands are considered to be Dutch [32], and persons who have at least one parent born in Morocco/Turkey are considered to be Moroccan/Turkish [33]. Applicants are excluded if they have any mental or physical disability that makes participation in a lifestyle intervention impossible, already participate in a regular vigorous exercise programme, or are pregnant or lactating.

#### Recruitment

Potential participants will be selected by GPs located in disadvantaged neighbourhoods or GPs who have many Turkish or Moroccan patients. GPs will make a selection of eligible patients in their database on the basis of the inclusion criteria regarding postal codes of deprived neighbourhoods [18], age and medication use. In addition, GPs will be asked to select the targeted ethnicities and to exclude those individuals who are unable to participate in the intervention because of their mental or physical condition. GPs will send an invitation to the selected patients to participate in the intervention study. The invitation will contain a brief screening questionnaire/registration form, an information booklet about the study, a tape measure and a return envelope. Participants of Turkish and Moroccan origin will receive the information materials in both Dutch and Turkish or Arabic, respectively.

In addition, multiple other recruitment strategies will be used. The intervention study will be promoted in the local community by researchers with the help and involvement of community health workers (e.g. social workers), local health professionals and other local contacts. Recruited participants will also be asked to inform friends or family members about the study and to ask them to participate in the study, if they meet the study criteria. Potential participants will fill in a screening questionnaire to check for the inclusion and exclusion criteria.

### Intervention group

The lifestyle programme will last for 12 months. It will consist of four group meetings, four hours of individual dietary advice and weekly sports lessons provided in the neighbourhood. The first group meeting is an introduction/kick-off meeting, guided by the researcher, in which participants get to know the dietician, the sports instructor and other study participants. The other three group meetings are about nutrition and are guided by the dietician. The topics of these meetings include comparing products/reading labels, dealing with social occasions and making affordable choices in the supermarket. The four hours of individual dietary advice will be divided over a flexible number of consultations in order to suit the needs of the participants. Participants will receive dietary advice from an ethnicity-matched dietician and information leaflets from the Netherlands Heart Foundation and the Netherlands Nutrition Centre on the benefits of healthy nutrition and increased physical activity. If these information leaflets are available in Turkish or Arabic, participants will be provided with leaflets in the language of their choice.

The physical activity lessons will be set up especially for the study participants. The lessons will be offered for men and women separately. Female and male sports instructors will be involved to provide gender-matched physical activity lessons. Participants will also be allowed to bring a friend or family member to increase social support.

Participants will receive the results of their anthropometric measurements, blood glucose and total cholesterol concentrations, and their physical activity levels after the baseline measurements as well as after the end measurements. The participants’ GPs will receive anthropometric values, blood values and urine values.

### Control group

At one group meeting, guided by a dietician, control group participants will receive general advice about a healthy diet. At the end of this meeting, participants will receive information leaflets on the benefits of healthy nutrition and increased physical activity. If these information leaflets are available in Turkish or Arabic, participants will be provided with leaflets in the language of their choice. Like in the intervention group, control group participants and their GPs will receive the results of the measurements.

### Measurements

The researcher will make an appointment with (potential) participants at a location close to their home to measure their anthropometrics and blood pressure. Subsequently, the participant will receive a referral letter for the medical laboratory in their neighbourhood to hand in a urine sample and to have blood taken for testing. In addition, participants will be asked to fill in several questionnaires. Participants can choose to complete these questionnaires in Dutch or in their mother tongue. They will be asked whether they prefer to fill in the questionnaires themselves at home or with a research assistant speaking their mother tongue. All measurements, except the process evaluation measures, will be performed at baseline and after 12 months.

#### Physical measurements

Blood samples will be taken after at least 10 hours of fasting to measure fasting glucose, HLD cholesterol, LDL cholesterol, total cholesterol, triglycerides, HbA1c, fasting insulin, liver function enzymes, creatinine and uric acid. Fasting spot urine samples will be collected to measure creatinine and microalbumin. Blood pressure will be measured six times, with two minutes rest in-between, in a seated position, with the Omron 705CP. The mean will be calculated from the last five measurements. Anthropometric measurements will be taken, including body weight, waist circumference, hip circumference, body fat percentage and height. Height will be measured to the nearest 0.1 cm. Waist circumference will be determined midway between the lowest rib and the iliac crest, and measured to the nearest 0.5 cm. Hip circumference will be measured to the nearest 0.5 cm at the widest portion of the buttocks. Waist and hip circumference will both be measured twice. Body weight and body fat percentage will be measured with the Tanita BC-418 (Tanita Corporation, Tokyo, Japan).

#### Physical activity

To evaluate changes in physical activity level, participants will fill in the validated Short QUestionaire to Assess Health enhancing physical activity (SQUASH) [34]. A question on sedentary behaviour was added based on the Activity Questionnaire for Adults and Adolescents (AQuAA) [35]. Participants will additionally wear an activity monitor (GT3X+ Actigraph, Pensacola, FL, USA) for seven days in order to measure physical activity.

#### Dietary intake

To evaluate changes in diet, an ethnic-specific Food Frequency Questionnaire (FFQ) will be administered [36].

#### Determinants of behaviour

A questionnaire has been developed to gain insight into determinants of behaviour. Questions to measure barriers to, and reasons for, healthy eating and physical activity are based on questions used in the Pan-EU Survey [37]. Items to measure perceived social influence are based on scales described by Schulz et al. [38]. The extent to which participants intend to be physically active and eat healthily will be assessed by the means of a Stages of Change Scale based on Prochaska and DiClemente’s Transtheoretical Model [39]. To assess knowledge with regard to nutrition, participants will be asked to select the healthiest choice from 10 pairs of products [40].

#### Quality of life

Quality of life will be assessed with the SF-36 questionnaire [41].

#### Process evaluation

A process evaluation guide has been developed on the basis of items described in the literature [42-46], including process evaluation measures to evaluate recruitment, reach, dose delivered, implementation integrity and programme acceptability. Data will be gathered by means of logbooks, registration forms, participants’ questionnaires, non-response survey, drop-out survey and individual interviews with the dieticians and sports instructors.

#### Economic evaluation

Costs and effects of the intervention programme will be compared with costs and effects of the control programme. The economic evaluation consists of a cost-effectiveness analysis and cost-utility analysis, and will be done from a societal and a healthcare perspective. A time horizon of 12 months will be used. Change in waist circumference will be used as clinical outcome for the cost-effectiveness analysis, and quality-adjusted life years (QALYs) for the cost-utility analysis. QALYs will be assessed with the EuroQoL instrument (EQ-5D-5L) [47,48]. Healthcare costs, patient costs and participants’ productivity losses will be assessed with a questionnaire. The intervention costs, including both staffing and materials, will be assessed on the basis of the attendance lists, registration forms and project logbooks of the health professionals and/or researchers. The Dutch guidelines for costing research within health economic evaluations will be used to value costs [49].

### Statistical analyses

For the effect evaluation, the intention-to-treat method will be followed. Changes in effect outcomes will be compared between the intervention and the control group. Analyses will be adjusted for age, gender, ethnicity and other possible confounders. For the process evaluation, both quantitative and qualitative data will be collected. Interviews will be analysed using a thematic approach. Quantitative data will be described by means and frequencies. Characteristics of the responders versus non-responders, and of the completers versus drop-outs, will be analysed by means of an independent sample *t*-test or chi-squared test. For the economic evaluation, the incremental cost-effectiveness ratio will be calculated on the basis of the differences in costs and effects between the intervention and the control programme. Bootstrapping will be used to calculate confidence intervals around costs and effects. A cost-effectiveness acceptability curve will be constructed from which it can be judged whether the intervention is cost-effective given a range of cost-effectiveness thresholds. The cost-effectiveness analyses will be complemented with sensitivity analyses for critical assumptions.

## Discussion

This article provides a detailed description of the MetSLIM study protocol, which is based on the SLIM study protocol. Furthermore, this article gives insight into the obstacles encountered in developing the MetSLIM study targeting low SES individuals of different ethnic origins. Adaptations to the original SLIM study protocol were considered necessary in order to overcome practical barriers that hinder the target group’s participation; to suit the (cultural) needs of the target group; and to make it feasible to perform the study in a local (community) setting. The main adaptations regarding the lifestyle programme, which will be offered to the intervention group, are: 1) additional group meetings about price concerns and social occasions with regard to a healthy diet; 2) ethnicity-matched dietician; 3) gender-matched sports instructor; 4) all activities in the participants’ own neighbourhood; and 5) activities for women and men separately. These adaptations are expected to be relevant for both the recruitment and retention of participants and for the successful delivery of the lifestyle programme [50].

A strict comparison between the effects of the adapted and the original lifestyle programme will be difficult. The target groups of SLIM and MetSLIM vary more than just in socioeconomic status and ethnicity. MetSLIM will include persons at a younger age and with an elevated waist circumference instead of impaired glucose tolerance, and excludes persons who use medication for cardiometabolic diseases. Consequently, the study population of the two studies could differ in health status, and this might influence both the interest in participating in a lifestyle programme, either positively or negatively [5,51], and the possible health gains from participating in a lifestyle programme [52].

A strength of the adaptation from the SLIM study protocol to the MetSLIM study protocol is that we involved the target group, (health) professionals and other researchers, and checked possibilities in the local setting while designing the MetSLIM study protocol. This enabled the creation of a study protocol that takes into account both the needs of the target group and what is actually possible in the local setting. In the end, however, researchers made the final decisions in the design of the MetSLIM study protocol. Although practically challenging, it could have been useful to involve the target group and health professionals in this decision making as well, in order to take into account the balance between evidence-based concerns and the acceptability or applicability of the intervention [50,53]. The multidisciplinary backgrounds of the research team, however, contributed to a careful consideration of the advantages and disadvantages of various choices in the study protocol. In addition, to check the applicability of several intervention materials, the materials were assessed by local health professionals and a communications expert.

The current article illustrates, next to a detailed description of the MetSLIM study protocol, several considerations that should be taken into account when a study protocol is being adapted or developed for individuals with low SES of different ethnic origins. Transparency, by sharing these considerations and choices made in the development of a study protocol, can help other researchers and health professionals to create appropriate strategies for (testing the effectiveness of) lifestyle interventions for this target group. Recruitment for the MetSLIM study started in January 2013 and data collection is expected to finish in June 2015. The process evaluation of the MetSLIM study will provide insight into the consequences of the choices made in the adapted study protocol in terms of reach, acceptability and delivery of the programme, and the effect and economic evaluation will provide insight into the (cost)effectiveness of the adapted lifestyle programme to reduce waist circumference among individuals with low SES of different ethnic origins.
